# Reprogramming Static Deformation Patterns in Mechanical Metamaterials

**DOI:** 10.3390/ma11102050

**Published:** 2018-10-20

**Authors:** Larry A. Danso, Eduard G. Karpov

**Affiliations:** Department of Civil & Materials Engineering, University of Illinois, Chicago, IL 60607, USA; lappia2@uic.edu

**Keywords:** transformation mechanics, mechanical metamaterials, lattice materials, static Raleigh waves, Saint Venant’s effect reversal, deformation reprogramming

## Abstract

This paper discusses an x-braced metamaterial lattice with the unusual property of exhibiting bandgaps in their deformation decay spectrum, and, hence, the capacity for reprogramming deformation patterns. The design of polarizing non-local lattice arising from the scenario of repeated zero eigenvalues of a system transfer matrix is also introduced. We develop a single mode fundamental solution for lattices with multiple degrees of freedom per node in the form of static Raleigh waves. These waves can be blocked at the material boundary when the solution is constructed with the polarization vectors of the bandgap. This single mode solution is used as a basis to build analytical displacement solutions for any applied essential and natural boundary condition. Subsequently, we address the bandgap design, leading to a comprehensive approach for predicting deformation pattern behavior within the interior of an x-braced plane lattice. Overall, we show that the stiffness parameter and unit-cell aspect ratio of the x-braced lattice can be tuned to completely block or filter static boundary deformations, and to reverse the dependence of deformation or strain energy decay parameter on the Raleigh wavenumber, a behavior known as the reverse Saint Venant’s edge effect (RSV). These findings could guide future research in engineering smart materials and structures with interesting functionalities, such as load pattern recognition, strain energy redistribution, and stress alleviation.

## 1. Introduction

Metamaterials is a term that has become synonymous with materials with unusual properties that enable exotic or anomalous functionalities, first advocated by Veselago in 1967 [1], in the context of physics of materials with negative permittivity and negative permeability, resulting in a negative refractive index. A validation of this fascinating theory by illustrative works, such as Pendry and Smith [2,3,4], has excited research on applications of photonic metamaterials [5,6,7,8,9], such as wave guiding and imaging resolution enhancement. The theory of negative refractive index has seen similar consequences in sound and wave mechanics when artificial materials are developed with a negative bulk modulus and negative mass density. The ability to control sound waves is therefore realized and the existence of bangaps in material dispersion curves can be utilized for sound and seismic wave insulation and reflection [10,11,12,13].

Lubenski, Bertoldi, and others have also studied topological metamaterials [14,15,16], lattice materials that are on the verge of mechanical instability due to their topological index and so exhibit zero-frequency modes or floppy modes. More interesting is how the release of internal stress in these lattices can lead to localization of those zero modes at a boundary surface without propagation. Such surface qualities allow for applications, such as adhesion and grip enhancement in rubber tires, as well as compliancy and energy absorption in rigid load bearing elements [17,18]. Other studies [19] on isostatic lattice network have been able to achieve any arbitrary global deformation by periodic actuation of substructure members. A recent study by Karpov [20] on the reverse Saint-Venant edge effect in periodic highly non-local lattice networks has also introduced the concept of deformation decay spectrum, where the presence of bandgaps in such a spectrum leads to blockage of an applied Raleigh mode deformation and the ability to reverse its decay rate. The existence of bandgaps and their effect on static Raleigh wave mode propagation in periodic lattices could be related to acoustic metamaterials and how their bandgap characteristics define dynamical wave propagation. The study [20], even though restricted to 1DoF lattices, is the first to provide an avenue to discuss how arbitrary modes of static deformation could be programmed in a periodic lattice network.

Our current interest is to enable programming of a 2D, nonlocal, x-braced lattice to manifest interesting functionalities, such as boundary deformation localization, deformation pattern recognition, strain energy redistribution, and stress and strain alleviation inside the lattice. The use of periodic material systems has showed a great importance in structural and materials engineering, where design and analysis are undertaken at the repetitive unit cell level to provide elegant approaches to cost-effective solutions. Geometrical periodicity has helped in analyzing many interesting micro-structural properties exhibited by metamaterials. Some of the earlier quasistatic analysis of patterned and repetitive structures involved the use of the discrete field analysis as a solution method for the governing system of finite difference equations [21,22]. A compact matrix form of these equations with a discrete convolution operator was also suggested [23], and discrete Fourier transform (DFT) methods were used to write computationally efficient semianalytical solutions for arbitrary force loads in terms of lattice Green’s function operators [24]. The Green’s function approach also allowed for various domain reduction techniques in molecular mechanics of materials, including single and multilayered graphene and carbon nanotubes [25,26,27,28].

Studies of static deformation in periodic lattices have been done in recent works [29,30,31,32] using several methods, such as the transfer matrix approach for 1D (beam like) structures. Using the transfer matrix approach, these authors [32] realized exponential decay of self-equilibrated end-load components, and rather simple polynomial behavior of tensile and bending displacement fields, even in complex architectural trusses and microstructured beams. Exponential decay of self-equilibrated sets of forces and couple moments at the beam ends was discussed as a manifest of the Saint-Venant principle in a discrete elastic media, and the corresponding decay rates were related to the transfer matrix eigenvalues.

Fully analytical solutions for discrete 2D materials and structures are interesting due to their immense technological significance realized in the recent decades. It was recently shown [20] that with a combination of the Fourier and transfer matrix methods we can produce a fundamental solution as a static surface mode or harmonic, which propagates in the material volume without any shape transformation. Only the amplitude of these modes decays exponentially at a known rate, λ<1, depending on the Fourier parameter, q∈(−π,π), termed the static Raleigh wave solution:

(1) $$d_{nm}=C\left(q\right)h\left(q\right)\lambda^{n}\left(q\right)e^{iqm}$$

Here, the index, *n*, increases toward the material interior; the index, *m*, varies along the side edges of a discrete two-dimensional material or structure; and $d_{nm}$ is a vector of all displacement components in any group of repetitive structural nodes numbered (*n*, *m*). C(q) is an arbitrary complex amplitude of the Raleigh wave, so that the real and imaginary parts of the solution (Equation (1)) separately represent two possible physical states of static deformation; the decay rate, *λ*(*q*), and polarization vector, **h**, are the eigenvalue and normalized half-eigenvector of a transfer matrix written in terms of partial Fourier images of lattice force constants, as explained in Section 2. 

A monotonous increase of *λ*(*q*) with *q* in Equation (1) is a manifest of the Saint-Venant principle in discrete 2D material systems, because *q* is a basic measure of unevenness of the Raleigh mode (Equation (1)), whose mean square deformation gradient over index, *m*, is proportional to $q^{2}$. Thus, any acute mode with a higher *q* should generally decay faster in the material volume than a smooth mode with a smaller *q*. However, anomalous behavior of the *λ*(*q*) dependence is possible, as was recently shown [20], where a group of vertical asymptotes (asymptotic bandgaps), and therefore negative slope intervals, can be introduced. This imply that certain small-scale unevenness may in fact prevail over coarse ones and propagate farer in the material interior. The ability of an engineering structural material to support anomalous propagation of fine-scale details of deformation in the material volume was named the reverse Saint-Venant’s effect (RSV) [20], and the material itself—the RSV metamaterial. Another practical significance of the asymptotic bandgap in static Fourier spectra of RSV metamaterials is the ability to completely detain certain types of deformation on the material surface, including some rather smooth ones.

A systematic understanding of static load processing and modification in RSV metamaterials require simple analytic and numerical tools for reconstruction and testing of various deformation patterns in the material volume for any type of practical natural and essential boundary conditions. In this paper, we outline a simple semianalytical approach to reconstruct analytical solutions for any essential (displacement) or natural (forcing) boundary condition applied at one edge of any nonlocal lattice, where the opposite edge is free and indefinitely remote, and periodic boundary conditions are applied at two other opposite edges. A bandgap design approach is also presented to provide a designer the flexibility to program the 2D x-braced lattice load bearing and strain energy storage capabilities, and to engineer lattice materials capable of blockage and polarization of particular Raleigh waves (Equation (1)) on the materials surface.

## 2. Displacement Transfer Matrix and Polarization Vectors

We start with the governing equation of equilibrium of an arbitrary nonlocal elastic medium with only pair-wise elastic interactions in the form of non-buckling bars, springs, or linearized interatomic bonds [20,24,26,27,28]:

(2) $${\left(k*d\right)}_{nm}=\sum_{n^{\prime }m^{\prime }}k_{n-n^{\prime }m-m^{\prime }}d_{n^{\prime }m^{\prime }}=f_{nm}$$

Vectors, $d_{nm}$ and $f_{nm}$, are comprised of all displacement and external force components in an arbitrary repetitive group of lattice nodes numbered (*n*, *m*). Matrices, **k**, represent the configuration and intensity of the elastic interactions between nodes of the current group and all its neighbor groups (*n’*, *m’*). The repetitive group is selected as large enough for the *n’*-summation to run from *n* − 1 to *n* + 1 only, while the *m*’-summation (along the lattice edge) may run for an arbitrary range. Structural periodicity implies dependence of **k** only on the differences *n-n’* and *m-m’*, rather than separate dependences on the current and running indices, which would be the case for non-periodic lattices. Assume that we know an essential boundary condition, $d_{0m}$, or a natural boundary condition, $f_{0m}$, on the lattice edge and we aim to determine the displacement solution, $d_{nm}$, where n>0. This solution will describe the state of free static deformation in the lattice interior, arising in response to a particular boundary condition.

For all n>0, we may write a homogenous governing equation:

(3) $$\sum_{n^{\prime }m^{\prime }}k_{n-n^{\prime }m-m^{\prime }}d_{n^{\prime }m^{\prime }}=0$$

Taking the summation over $n^{\prime }$ in Equation (3) from *n* − 1 to *n* + 1, we may write:

(4) $$\sum_{m^{\prime }}k_{1\;m-m^{\prime }}d_{n-1\;m^{\prime }}+k_{0\;m-m^{\prime }}d_{n\;m^{\prime }}+k_{-1\;m-m^{\prime }}d_{n+1\;m^{\prime }}=0$$

The Fourier domain form of Equation (4) is obtained after performing the discrete Fourier transform (DFT) over its terms to get:

(5) $$K_{1\;}\left(q\right)d_{n-1\;}\left(q\right)+K_{0\;}\left(q\right)d_{n\;}\left(q\right)+K_{-1\;}\left(q\right)d_{n+1\;}\left(q\right)=0$$

where $K_{n\;}\left(q\right)=\sum_{m}k_{nm}e^{-iqm}$ and $d_{n}\left(q\right)=C\left(q\right)h\left(q\right)\lambda^{n}\left(q\right)$, according to Equation (1). Hence, the displacement transfer matrix, H(q), is formulated from Equation (5) as:

(6) $$H\left(q\right)\left\{\begin{array}{c} d_{n-1\;}\left(q\right)\\ d_{n\;}\left(q\right) \end{array}\right\}=\left\{\begin{array}{c} d_{n\;}\left(q\right)\\ d_{n+1\;}\left(q\right) \end{array}\right\}$$

$$H\left(q\right)=\left[\begin{array}{cc} 0 & I\\ -K_{-1}{\left(q\right)}^{-1}K_{1}\left(q\right) & -K_{-1}{\left(q\right)}^{-1}K_{0}\left(q\right) \end{array}\right]$$

The static Raleigh wave mode solution in Equation (1), as mentioned earlier, is dependent on the eigensystem of matrix, H(q), since the size of this matrix is 2*R* × 2*R* where *R* is the number of degrees of freedom.

**Figure 1:**
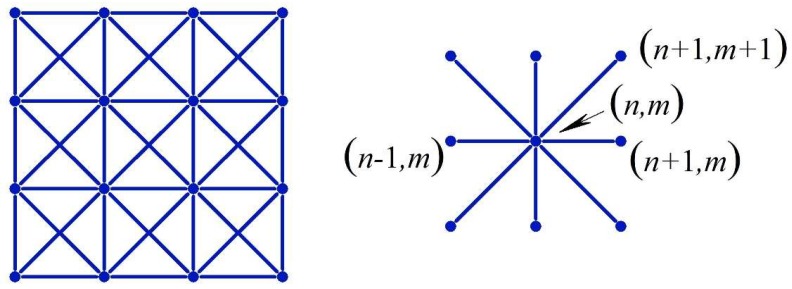
A fragment of the periodic x-braced lattice, and its associated substructure.

At a repetitive group of lattice nodes, there will be 2*R* eigenvalues, *λ*(q). However, these eigenvalues will come in reciprocal pairs as a consequence of the symplectic [33,34,35] nature of the displacement transfer matrix, H(q). If λ(q) is an eigenvalue of **H**(*q*), then 1/λ(q) is also an eigenvalue, and they could be either a real or complex conjugate pair. For a convergent physical solution (Equation (1)), we select only *R* eigenvalues, such that |λ(q)|≤1. The 2*R*-component eigenvectors will have the form $\left\{\begin{array}{c} h\left(q\right)\\ \lambda \left(q\right)h\left(q\right) \end{array}\right\}$, where the bottom half-vector is the top half-vector multiplied by λ(q) [20]. Considering an x-braced lattice with two degrees of freedom per node, see Figure 1, the 4 × 4 transfer matrix, H(q), is consistent with the structure:

(7) $$H\left(q\right)=\left[\begin{array}{cccc} 0 & 0 & 1 & 0\\ 0 & 0 & 0 & 1\\ \beta_{1} & \beta_{3}i & \beta_{4} & \beta_{6}i\\ \beta_{2}i & \beta_{1} & \beta_{5}i & \beta_{7} \end{array}\right]$$

$$\beta_{1}=-\frac{\left(\sqrt{2\;}\cos q+k\cos2q\right)}{k+\sqrt{2}\cos q},\;\;\beta_{2}=\frac{2\left(\sqrt{2\;}+k\cos q\right)\sin q}{k+\sqrt{2}\cos q},\;\;\beta_{3}=\frac{k\;\sin q}{k+\sqrt{2}\cos q},\;\;\beta_{4}=\frac{2\left(\sqrt{2\;}+k\right)\cos q}{k+\sqrt{2}\cos q}$$

$$\beta_{5}=-\frac{2\left(\sqrt{2}+k\right)\sin q}{k+\sqrt{2}\cos q},\;\;\beta_{6}=-\frac{2\left(k-\sqrt{2\;}\left(k\cos q-1\right)\right)\sin q}{k+\sqrt{2}\cos q},\;\;\beta_{7}=\frac{\left(2+k\sqrt{2}-2\cos q\right)\left(2+\sqrt{2\;}k\cos q\right)}{k+\sqrt{2}\cos q}$$

where *k* represents the relative stiffness of the diagonal bars over vertical or horizontal bars. The vertical and horizontal bars have the same stiffness. Solving the eigenvalue problem, $\left(H\left(q\right)-I\lambda \left(q\right)\right)\left\{\begin{array}{c} h\left(q\right)\\ \lambda \left(q\right)h\left(q\right) \end{array}\right\}=0$, the eigenvector is derived Equations (A1)–(A4) in a compact form as:

(8) $$\left\{\begin{array}{c} h\left(q\right)\\ \lambda \left(q\right)h\left(q\right) \end{array}\right\}=C\left(q\right)\left\{\begin{array}{c} \begin{array}{c} i\left(\beta_{3}+\beta_{6}\lambda \right)\\ \lambda^{2}-\beta_{4}\lambda -\beta_{1}\\ i\;\lambda \left(\beta_{3}+\beta_{6}\lambda \right) \end{array}\\ \lambda \left(\lambda^{2}-\beta_{4}\lambda -\beta_{1}\right) \end{array}\right\}$$

Here, C(q) could be any real or complex number for a given value, q, and this number is chosen to normalize the half-eigenvectors, so that |h(q)|=1 at all q. It can be seen in Equation (8) that the bottom half-of the eigenvector is equal to the top half-vector multiplied by the eigenvalue, λ(q). The polarization vector for a complex eigenvalue can be derived by substituting the complex conjugate eigenvalue pair, λ(q)=μ±ωi, into the top half-vector of Equation (8) and regrouping of the real and imaginary parts to get:

(9) $$h\left(q\right)=\left\{\begin{array}{c} a\pm ib\\ -c+id \end{array}\right\}$$

$$\;a=-\beta_{6}\omega ,\;\;b=\left(\beta_{3}+\beta_{6}\mu \right),c=\left(-\beta_{1}-\beta_{4}\mu +\mu^{2}-\omega^{2}\right),\;\;d=\left(2\mu \omega -\beta_{4}\omega \right)$$

The polarization vector in the instance of a real eigenvalue, λ(q), is derived directly from the polarization vector, **h**(*q*), of the complex eigenvalue by eliminating the imaginary part, ω, in Equation (9) to obtain the form:

(10) $$h\left(q\right)=\left\{\begin{array}{c} ib\\ c \end{array}\right\}$$

## 3. 2DoF Static Raleigh Wave Solution

Since the Raleigh mode solution is being built for a periodic spatial domain along the index, *m,* of a lattice, the static Raleigh wave solution (Equation (1)) must be constructed such that it is a real-valued cyclic solution by obeying the following symmetry condition about the mid-plane of a periodic domain:

(11) $$d_{nm}=\left\{\begin{array}{c} U_{nm}\\ V_{nm} \end{array}\right\}=\left\{\begin{array}{c} U_{nm}\\ -V_{nm} \end{array}\right\}$$

Constructing $d_{nm}$, we first substitute λ(q) and **h**(*q*) into Equation (1) and obtain a solution containing real and imaginary parts. Basically, that part of the solution satisfying (11) is considered the real-cyclic solution. Should the eigenvalues be complex-valued, λ(q)=μ±ωi, it would be convenient to utilize the polar coordinate form, $\lambda \left(q\right)=\rho e^{i\theta }$, where ρ=|λ(q)| and θ=Arg(λ(q)). Since eigenvalues are complex conjugate, after substituting them into $d_{nm}$, we obtain four (4) possible solutions (two real parts and two imaginary parts), although not satisfying the symmetry condition Equation (11). However, we can reduce this to the solution in Equation (12) that satisfies the condition in Equation (11) by summing the two real parts and subtracting the two imaginary parts. Below, we state the real-valued cyclic solution forms Equations (A5)–(A12) for the complex and real eigenvalues as:

*Complex Eigenvalue:*

(12) $$h\left(q\right)=\left\{\begin{array}{c} a\pm ib\\ -c+id \end{array}\right\}:\;\;d_{nm}=\{\begin{array}{c} C_{1}\rho^{n}\left(q\right)\left\{\begin{array}{c} a\;\cos\;qm\\ -d\;\sin\;qm \end{array}\right\}\\ C_{1}\rho^{n}\left(q\right)\left\{\begin{array}{c} b\;\cos\;qm\\ c\;\sin\;qm \end{array}\right\} \end{array}$$

*Real Eigenvalue:*

(13) $$h\left(q\right)=\left\{\begin{array}{c} ib\\ c \end{array}\right\}:\;\;d_{nm}=C_{2}\lambda^{n}\left(q\right)\left\{\begin{array}{c} b\;\cos\;qm\\ c\;\sin\;qm \end{array}\right\}$$

In Equations (12) and (13), the coefficients (a, b, c, d) reconstruct the polarization vector, h(q), of the Fourier parameter, *q*, that constructs real-cyclic Raleigh mode solution in Equation (1). 

## 4. Arbitrary Essential Boundary Condition Solution

Our discussion till now has been concerned with static Raleigh wave solution forms, which are essentially harmonic (cosine, sine), but in practice, applied boundary conditions could be in several forms, such as a gaussian, impact, triangular, etc., and therefore this section is dedicated to finding a solution form that would satisfy an arbitrary essential boundary condition, $d_{0m}$. Considering Equation (1), a general solution is obtained by the summation of all possible modes of the decomposed essential boundary condition as:

(14) $$d_{nm}=\frac{1}{M}\sum_{q=0}^{M-1}\left[\begin{array}{cc} h_{1}\left(q\right) & h_{2}\left(q\right) \end{array}\right]{\left[\begin{array}{cc} \lambda_{1}\left(q\right) & 0\\ 0 & \lambda_{2}\left(q\right) \end{array}\right]}^{n}\left\{\begin{array}{c} C_{1}\left(q\right)\\ C_{2}\left(q\right) \end{array}\right\}e^{iqm}$$

where $\left[\begin{array}{cc} h_{1}\left(q\right) & h_{2}\left(q\right) \end{array}\right]$ is a matrix of the column-vector components. In Equation (14), $C_{1}\left(q\right)$ and $C_{2}\left(q\right)$ are some Fourier coefficients to be determined from boundary conditions. Considering an arbitrary boundary deformation, $d_{0m}$, a semi-analytical procedure is developed for finding $C_{1}$ and $C_{2}$ by multiplying both sides of Equation (14) with the conjugate transpose of a normalized polarization vector, $h_{1}^{*}\left(q^{\prime }\right)$, and performing the DFT on both sides of the same equation.

(15) $$\frac{1}{M}\sum_{m=0}^{M-1}h_{1}^{*}\left(q^{\prime }\right)d_{0m}e^{-iq^{\prime }m}=\frac{1}{M^{2}}\sum_{m=0}^{M-1}\sum_{q=0}^{M-1}\left\{\begin{array}{cc} h_{1}^{*}\left(q^{\prime }\right)h_{1}\left(q\right) & h_{1}^{*}\left(q^{\prime }\right)h_{2}\left(q\right) \end{array}\right\}\left\{\begin{array}{c} C_{1}\left(q\right)\\ C_{2}\left(q\right) \end{array}\right\}e^{iqm}e^{-iq^{\prime }m}$$

Rearranging,

(16) $$\sum_{m=0}^{M-1}h_{1}^{*}\left(q^{\prime }\right)d_{0m}e^{-iq^{\prime }m}=\frac{1}{M}\sum_{q=0}^{M-1}\left(\sum_{m=0}^{M-1}e^{-iq^{\prime }m}e^{iqm}\right)\left\{\begin{array}{cc} h_{1}^{*}\left(q^{\prime }\right)h_{1}\left(q\right) & h_{1}^{*}\left(q^{\prime }\right)h_{2}\left(q\right) \end{array}\right\}\left\{\begin{array}{c} C_{1}\left(q\right)\\ C_{2}\left(q\right) \end{array}\right\}$$

Since,

(17) $$\sum_{m=0}^{M-1}e^{-iq^{\prime }m}e^{iqm}=M\delta_{qq^{\prime }}$$

Equation (16) can be simplified as:

(18) $$\sum_{m=0}^{M-1}h_{1}^{*}\left(q\right)d_{0m}e^{-iqm}=\left\{1\;h_{1}^{*}\left(q\right)h_{2}\left(q\right)\right\}\left\{\begin{array}{c} C_{1}\left(q\right)\\ C_{2}\left(q\right) \end{array}\right\}$$

Repeating the above procedure with $h_{2}^{*}\left(q^{\prime }\right)$, we obtain a similar expression as in Equation (18) and solve for $C_{1}\left(q\right)$ and $C_{2}\left(q\right)$ as:

(19) $$\left\{\begin{array}{c} C_{1}\left(q\right)\\ C_{2}\left(q\right) \end{array}\right\}={\left[\begin{array}{cc} 1 & h^{*}{}_{2}\left(q\right)h_{1}\left(q\right)\\ h^{*}{}_{1}\left(q\right)h_{2}\left(q\right) & 1 \end{array}\right]}^{-1}\left[\begin{array}{c} h_{1}^{*}\left(q\right)\\ h_{2}^{*}\left(q\right) \end{array}\right]\;d_{0}\left(q\right)$$

where $d_{0}\left(q\right)=\sum_{m=0}^{M-1}d_{0m}e^{-iqm}$ represents the DFT of the arbitrary essential boundary condition, and substituting $C_{1}\left(q\right)$ and $C_{2}\left(q\right)$ into Equation (14), a general solution is obtained that can fully represent static deformation in the lattice interior.

## 5. Natural Boundary Condition Solution

Having formulated a general solution form for analyzing arbitrary essential boundary conditions, it would be appropriate to deal with scenarios of a natural (forced) boundary condition, which has a greater practical appeal. Considering the Fourier form of the equilibrium governing Equation (4), at n=0, we can write:

(20) $$\frac{1}{2}K_{0}\left(q\right)d_{0}\left(q\right)+K_{-1}\left(q\right)d_{1}\left(q\right)=f_{0}\left(q\right)$$

where $f_{0}\left(q\right)=\sum_{m=0}^{M-1}f_{0m}e^{-iqm}$ represents the arbitrary natural boundary condition and Equation (20) represents the effect of neglecting all the nodal set to the left of the boundary where the natural boundary condition is applied and ignoring boundary stiffness interaction with the same by having the term, $\frac{1}{2}K_{0}\left(q\right)$, in Equation (20). Decomposing $d_{0}\left(q\right)$ and $d_{1}\left(q\right)$ from Equation (20) into their Fourier components and solving for $C_{1}\left(q\right)$ and $C_{2}\left(q\right)$, we get:

(21) $$\left\{\begin{array}{c} C_{1}\left(q\right)\\ C_{2}\left(q\right) \end{array}\right\}={\left[\frac{1}{2}K_{0}\left(q\right)\left\{\begin{array}{c} h_{1}\left(q\right)\\ h_{2}\left(q\right) \end{array}\right\}+K_{-1}\left(q\right)\left[\begin{array}{cc} h_{1}\left(q\right) & h_{2}\left(q\right) \end{array}\right]\left[\begin{array}{cc} \lambda_{1}\left(q\right) & 0\\ 0 & \lambda_{2}\left(q\right) \end{array}\right]\right]}^{-1}f_{0}\left(q\right)$$

Substituting $C_{1}\left(q\right)$ and $C_{2}\left(q\right)$ into Equation (14) and adding $G\left(n\right)=n\;K_{-1}{\left(0\right)}^{-1}{\tilde{f}}_{0}\left(q\right)$, a linear polynomial term is constructed to account for uniform deformation [32], after considering the possible canonical modes of static deformation of the Jordan block of the displacement transfer matrix, H(q). Hence, the general solution for any natural boundary condition is stated as:

(22) $$d_{nm}=\frac{1}{M}\sum_{q=0}^{M-1}\left[\begin{array}{cc} h_{1}\left(q\right) & h_{2}\left(q\right) \end{array}\right]{\left[\begin{array}{cc} \lambda_{1}\left(q\right) & 0\\ 0 & \lambda_{2}\left(q\right) \end{array}\right]}^{n}\left\{\begin{array}{c} C_{1}\left(q\right)\\ C_{2}\left(q\right) \end{array}\right\}e^{iqm}+G\left(n\right)$$

## 6. Raleigh Wave Mode Bandgap Design

Boundary deformation blockage or localization relates to the feature of asymptotic bandgaps in the deformation decay spectrum [20] for a periodic lattice structure, where *q* corresponds with a zero eigenvalue (λ=0) and, subsequently, the RSV effect, since η(q)=−logλ(q) starts to decrease in value as we increase *q*: Growth in the fineness of the static Raleigh wave mode corresponding to a slower deformation decay. The deformation decay spectrum is a map of the distribution of the decay parameter, η(q), over *q*; examples are shown in Section 8. The aim of this section is to develop a relationship between *k* and *q* for finding polarization vectors, h(q), that correspond to a bandgap in the lattice deformation decay spectrum. Knowing that H(q) is a square matrix, it is valid that its determinant is equal to the product of its eigenvalues, $\det H\left(q\right)=\prod_{i=1}^{n}\lambda_{i}\left(q\right)$, according to Vieta’s rule and so applying this property, a condition for attaining a zero eigenvalue, λ(q)=0, could be stated as when detH(q)=0. Applying this condition, a zero-eigenvalue relationship, Equations (A13) and (A14), is derived for a 2DoF x-braced lattice as:

(23) $$k+\sqrt{2}\cos q=0$$

**Figure 2:**
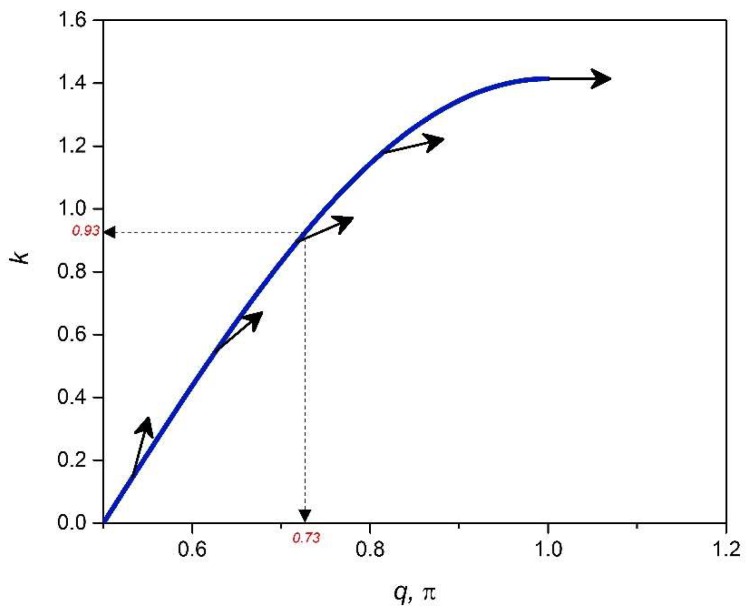
Occurrence of zero eigenvalues in the (*q*,*k*)-parameter space of Figure 1 lattice. Arrows represent the orientation of their corresponding polarization vectors, **h**(*q*).

Figure 2 shows a plot of *k* against *q* from Equation (23). This plot prescribes the stiffness parameter, *k,* of a 2DoF x-braced lattice that can be designed as a deformation blocker or filter, and the dark arrows on the plot describe the direction of polarization vectors, **h**(*q*), that would be arrested at the boundary surface of the prescribed x-braced lattice.

It is also possible to create a bandgap phase diagram for design purposes by introducing an aspect ratio, α, as a system parameter Equation (A15). In such a scenario, Equation (23) has the form:

(24) $$2k\alpha^{3}\sqrt{1+\alpha^{2}}+\cos q+2\alpha^{2}\cos q+\alpha^{4}H\cos q=0\;\;\alpha =\frac{\mathrm{breadth}}{\mathrm{heigth}}$$

**Figure 3:**
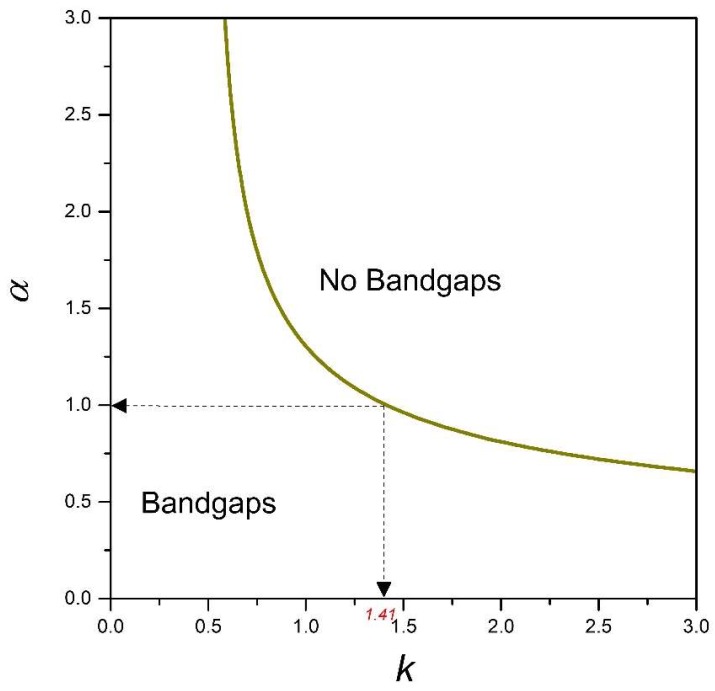
A phase diagram of the lattice material of Figure 1.

A plot of α against *k* from Equation (24), as shown in Figure 3, represents a design map for generating bandgaps when modelling a 2DoF x-braced periodic lattice. It also shows the range of *k* permissible in the design for a specific α. A typical x-brace lattice shown in Figure 1, with α=1, from Figure 3 has *k* ranging from 0–1.41, which was also seen in Figure 2. Figure 3 also shows that when α is between 0 and 0.5, there is no restriction on the parameter, *k*, for which a bandgap would exist. 

## 7. Polarizing Structures: Case of Repeated Zero Eigenvalues ($\lambda_{1}\&\lambda_{2}\to 0$)

In the above section, we dealt with boundary displacement blockage because of bandgap existence in the deformation decay spectrum of a lattice structure, where in the analysis of 2DoF systems, we required only a single λ=0 to reprogram a lattice to possess this displacement blocking feature. However, a case of repeated zero eigenvalues, where $\lambda_{1}=0$ and $\lambda_{2}=0$, presents a unique class of structures that could be termed as polarizers, with the mechanical property of reprogramming an arbitrary vector of a Raleigh deformation mode at n=0 into a desired polarization vector, h(q), at n=1 that would be completely blocked by the lattice structure at that point (n=1). Implementing a numerical searching procedure, it is possible to obtain repeated zero eigenvalues, but for a 2DoF lattice structure, the system transfer matrix, H(q), generates only a single independent eigenvector, $\left\{\begin{array}{c} h\left(q\right)\\ \lambda \left(q\right)h\left(q\right) \end{array}\right\}$, instead of two. Such a structure is deemed to have two (Equation (2)) modes of static deformation according to the Jordan canonical form of H(q) [32], written as:

(25) $$J=\left[\begin{array}{cc} 0 & 1\\ 0 & 0 \end{array}\right]$$

The two possible solution modes, $j_{n}^{\left(1\right)}$ and $j_{n}^{\left(2\right)}$, resulting from the above Jordan block would have the forms:

(26) $$j_{n}^{\left(1\right)}=0^{n}h$$

(27) $$j_{n}^{\left(2\right)}=0^{n}g+n0^{n-1}h$$

where h is the only independent eigenvector and g is the subsequently obtained generalized eigenvector. In the case of a 2DoF system with repeated zero eigenvalues, when the polarization vector, h(q), of the lattice structure is applied, we observe that the mode, $j_{n}^{\left(1\right)}$, controls the Raleigh mode static deformation, and displacements are blocked at n=0. Applying an arbitrary vector in Equation (1), we note that $j_{n}^{\left(2\right)}$, a combination of an exponential and polynomial modes, control static deformation in the lattice and displacements are blocked at n=1 instead, as shown below:

At n=1,

(28) $$j_{1}^{\left(2\right)}=0\;g+h=h$$

At n=2,

(29) $$j_{2}^{\left(2\right)}=0\;g+2\times 0\;h=0$$

From the above analysis, we can state that a lattice structure yielding repeated zero eigenvalue polarizes an arbitrary Raleigh mode at the position, n=1, and this wave completely disappears at n=2.

## 8. Illustrative Examples

**Figure 4:**
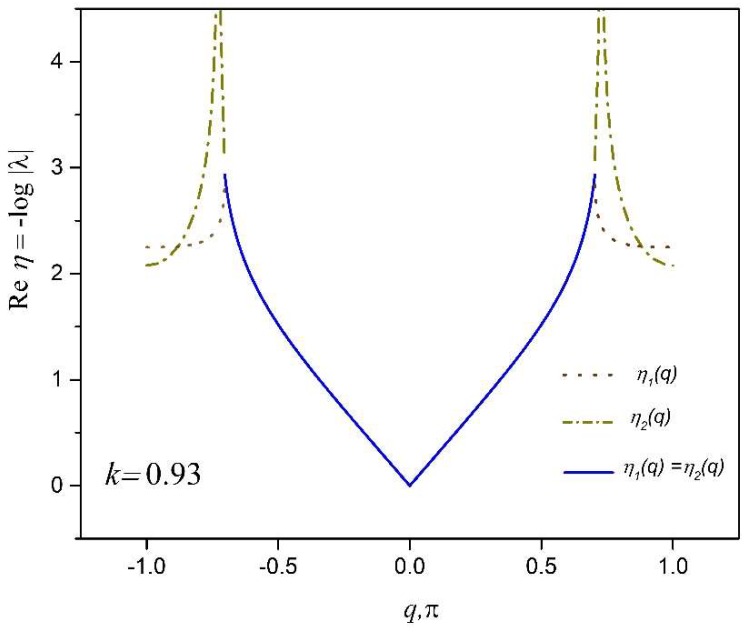
Deformation decay spectrum for an x-braced lattice (Figure 1) at k=0.93.

We begin our illustrations with several examples of the 2DoF x-braced lattice (Figure 1), with a relative stiffness parameter, k=0.93, and a deformation decay spectrum of Figure 4. First we consider a Raleigh mode solution (Equation (1)) for a Fourier parameter, $q=\frac{4}{5}\pi$, where the eigenvalues are $\lambda_{1}=0.10$ and $\lambda_{2}=-0.06$ and their corresponding eigenvectors are $h_{1}=\left\{\begin{array}{c} 0.9335\\ -0.3442i \end{array}\right\}$ and $h_{2}=\left\{\begin{array}{c} 0.4870i\\ 0.8710 \end{array}\right\}$. Since these eigenvalues are real, the real-valued cyclic static Raleigh wave solutions are to be calculated using Equation (13), as following:

(30) $$d_{nm}^{\left(1\right)}=C_{2}\lambda_{1}^{n}\left(q\right)\left\{\begin{array}{c} b\;\cos\;qm\\ -c\;\sin\;qm \end{array}\right\}=0.10^{n}\left\{\begin{array}{c} 0.9335\;\cos\frac{4}{5}\pi m\\ 0.3442\;\sin\frac{4}{5}\pi m \end{array}\right\}$$

(31) $$d_{nm}^{\left(2\right)}=C_{2}\lambda_{2}^{n}\left(q\right)\left\{\begin{array}{c} b\;\cos\;qm\\ c\;\sin\;qm \end{array}\right\}={\left(-0.06\right)}^{n}\left\{\begin{array}{c} 0.4870\;\cos\frac{4}{5}\pi m\\ 0.8710\;\sin\frac{4}{5}\pi m \end{array}\right\}$$

**Figure 5:**
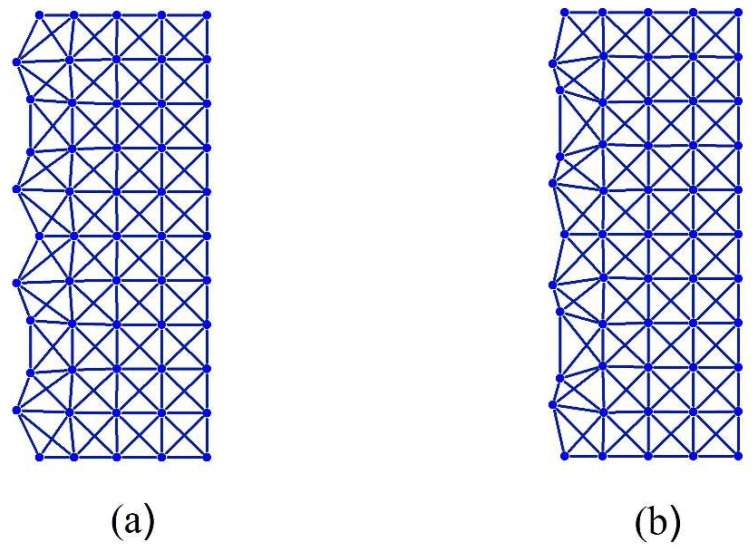
Deformation configuration (scaled) of the 2DoF x-braced lattice (k=0.93, $q=\frac{4}{5}\pi$, m=0→10, n=0→4) given by the analytical Equations (30) and (31) in (**a**) and (**b**), respectively.

Figure 5 shows the deformation configuration of the Raleigh mode solutions in Equations (30)–(31) for a vertical lattice dimension, *M* = 10. Now, taking $q=\frac{1}{5}\pi$ for the same stiffness parameter, k=0.93, a pair of complex conjugate eigenvalue, λ=0.5439±0.1425i, is obtained and the possible real cyclic solutions are constructed from Equation (12) as:

$$h=\left\{\begin{array}{c} 0.7537\pm 0.4554i\\ -0.8903-0.6572i \end{array}\right\}:$$

(32) $$d_{nm}^{\left(1\right)}=C\rho^{n}\left(q\right)\left\{\begin{array}{c} a\;\cos\;qm\\ -d\;\sin\;qm \end{array}\right\}=0.32^{n}\left\{\begin{array}{c} 0.7537\;\cos\frac{1}{5}\pi m\\ 0.6572\;\sin\frac{1}{5}\pi m \end{array}\right\}$$

(33) $$d_{nm}^{\left(2\right)}=C\rho^{n}\left(q\right)\left\{\begin{array}{c} b\;\cos\;qm\\ c\;\sin\;qm \end{array}\right\}=0.32^{n}\left\{\begin{array}{c} 0.4554\;\cos\frac{1}{5}\pi m\\ -0.8903\;\sin\frac{1}{5}\pi m \end{array}\right\}$$

**Figure 6:**
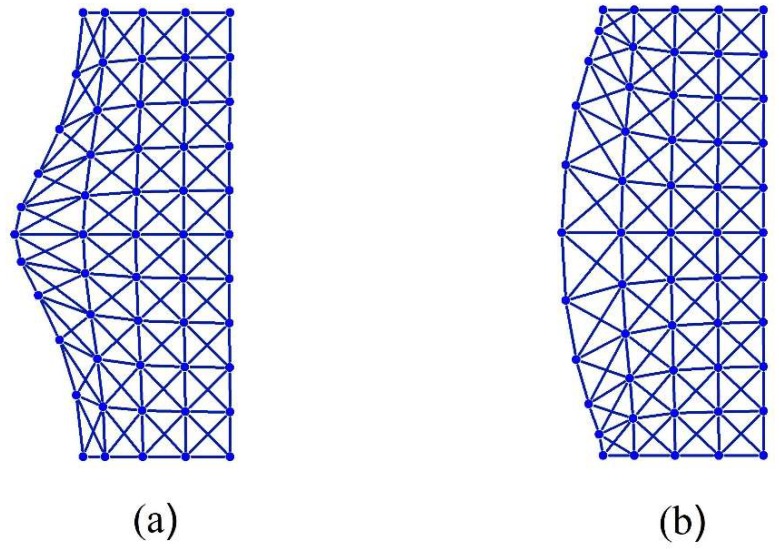
Deformation configuration (scaled) of the 2DoF x-braced lattice (k=0.93, $q=\frac{1}{5}\pi$, m=0→10, n=0→4) given by the analytical solutions (Equations (32) and (33)) in (**a**) and (**b**), respectively.

Figure 6 also shows the lattice deformed configuration corresponding to the Raleigh mode solutions of the Equations (32) and (33) for a lattice vertical dimension, *M* = 10.

Now, we would like to program an x-braced lattice having a deformation decay spectrum shown in Figure 4 to observe a block in Raleigh mode propagation and the RSV effect. Since the bandgap ($\lambda_{2}=0$) in Figure 4 exists at $q=\frac{8\pi }{11}\approx 0.73$, the RSV effect must be seen in the subsequent Fourier parameters and so we consider the Fourier parameters, $q=\frac{9\pi }{11}$ and $\frac{10\pi }{11}$. The Raleigh mode solutions for all three cases are constructed as:

(34) $$q=\frac{8\pi }{11}:\;\;d_{nm}=0.0015^{n}\left\{\begin{array}{c} 0.6609\;\cos\frac{8}{11}\pi m\\ 0.7504\;\sin\frac{8}{11}\pi m \end{array}\right\}$$

(35) $$q=\frac{9\pi }{11}:\;\;d_{nm}=-0.0756^{n}\left\{\begin{array}{c} 0.4495\;\cos\frac{9}{11}\pi m\\ 0.8901\;\sin\frac{9}{11}\pi m \end{array}\right\}$$

(36) $$q=\frac{10\pi }{11}:\;\;d_{nm}=0.1039^{n}\left\{\begin{array}{c} 0.9823\;\cos\frac{10}{11}\pi m\\ 0.1524\;\sin\frac{10}{11}\pi m \end{array}\right\}$$

**Figure 7:**
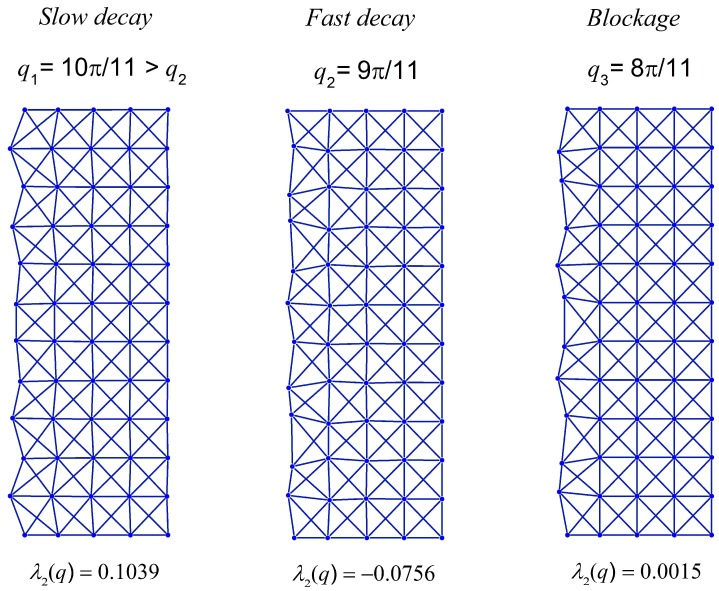
Deformation configuration (scaled) of the analytical solutions (Equations (34)–(36)), respectively, for half-cyclic domain of an x-braced lattice (k=0.93, m=0→11, n=0→4).

**Figure 8:**
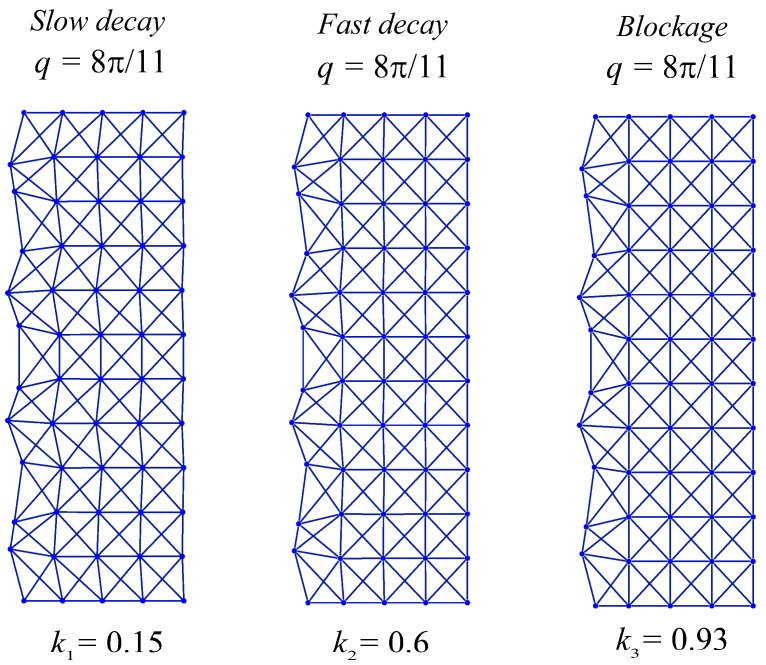
Deformation configurations (scaled) of different stiffness parameters under a Raleigh mode solution for $q=\frac{8\pi }{11}$ in a half-cyclic domain of the x-braced lattice (m=0→11, n=0→4 ).

The Raleigh mode deformation configurations of Equations (34)–(36) for a lattice vertical dimension, *M* = 22, are as shown in Figure 7. This figure shows a complete blockage of the Raleigh wave mode at $=\frac{8\pi }{11}$, as expected, and comparing the finer modes, $q=\frac{10\pi }{11}$, with the coarser mode, $q=\frac{9\pi }{11}$, we observe the RSV edge effect: The coarser mode decays faster than a finer one. In Figure 8, the deformation configurations for the Raleigh mode of $q=\frac{8\pi }{11}$ applied at n=0 are analyzed by varying the stiffness parameter, k. The ability to reprogram the x-braced lattice for blockage is realized as shown in Figure 8 by tuning k to the value corresponding to the band gap, which is $k_{3}=0.93$. We also observe how the rate of Raleigh mode decay in the lattice interior is programmed by tuning the stiffness parameter, where at $k_{1}=0.15$, we observe a slow Raleigh mode decay, and at $k_{2}=0.6$, a much faster decay occurs.

**Figure 9:**
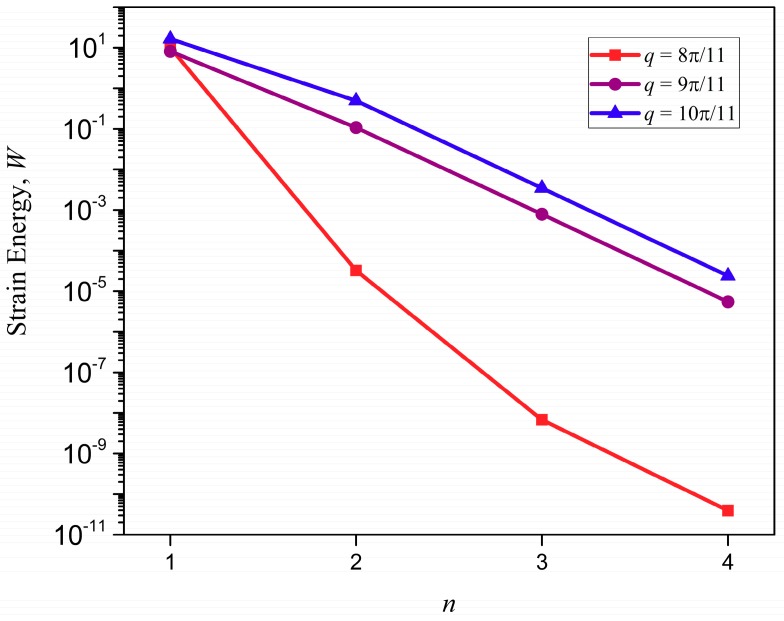
Strain energy along the index, *n*, in an x-braced lattice with *k* = 0.93: The fastest total strain energy decay occurs at $q=\frac{8}{11}\pi$, followed by $q=\frac{9}{11}\pi$, and then $q=\frac{10}{11}\pi$ due to the reverse Saint-Venant’s effect.

**Figure 10:**
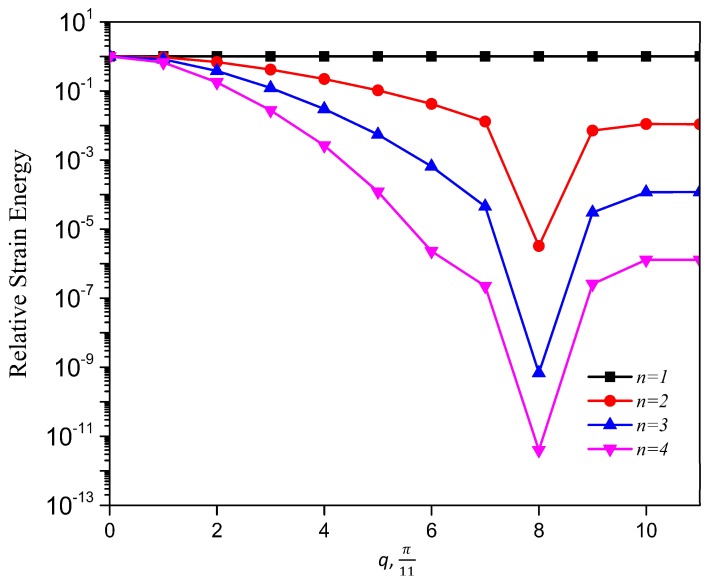
Relative strain energy against the Fourier parameter, *q*. A plot of strain energy at *n* relative to strain energy at *n* =1 against the Fourier parameter, *q*.

The distribution of strain energy inside a lattice, when mapped, could show interesting features and a spectrum of such a distribution could help when programming a lattice to harness the functionalities, such as strain energy redistribution and resilience in design. The total strain energy per vertical layer of a unit cell thickness is calculated by summing the strain energy stored at each associate substructure, Equations (A16) and (A17), over the lattice index, *m*, at a given horizontal index, *n*, in the x-braced lattice (Figure 1) is plotted against the value, *n*, for the example in Figure 7. Figure 9 shows that strain energy stored along the lattice index, *n*, follows similar trends as the decay of deformation along the index, *n*. Namely, the plot of energy for the mode, $q=\frac{8\pi }{11}$, exhibits the fastest decay followed by $q=\frac{9\pi }{11}$ and then $q=\frac{10\pi }{11}$, due to the RSV effect. To present a comprehensive picture to show that the pattern of strain energy decay in a lattice is analogous to its deformation decay, we also plot the strain energy along index, *n* (1→4), relative to n=1 for all possible static Raleigh modes in Figure 10. This figure shows a monotonous decay of the strain energy, as the parameter, *q*, of the Raleigh mode increases from 0 to $\frac{7\pi }{11}$. When *q* is $\frac{8\pi }{11}$, there is a much faster decay due to a bandgap (where λ=0) and after this point, as we increase *q,* the normalized strain energy at each lattice index, *n*, starts to increase, which manifests the RSV behavior.

**Figure 11:**
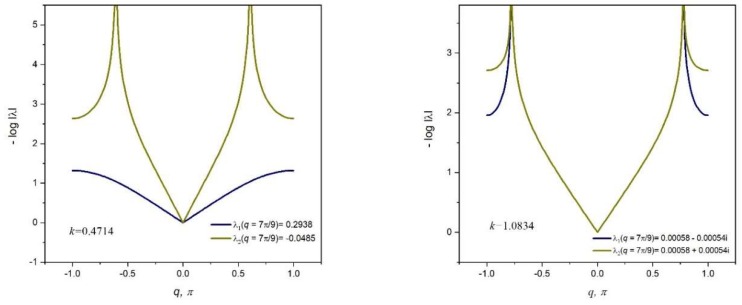
Deformation decay spectrum for *k* = 0.4714 and *k* = 1.0834, respectively.

At this instance, we illustrate the behavior of a polarizing lattice structure by analyzing two x-braced lattices of equal spatial dimensions; the first lattice with *k* = 0.4714, and the second lattice with *k* = 1.0834. The deformation decay spectrums of these two lattices are shown in Figure 11a,b, respectively.

**Figure 12:**
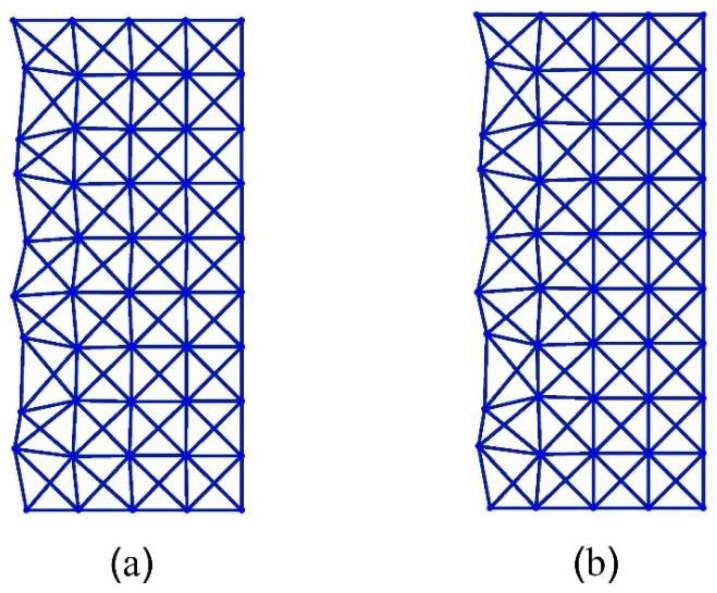
Deformation configurations (scaled) for a half-cyclic domain of the x-braced lattice (m=0→9, n=0→4 ) at *k* = 0.4714 (**a**), and *k* = 1.0834 (**b**).

From Figure 11, it can be referenced that at $q=\frac{7}{9}\pi$, the first x-braced lattice (*k* = 0.4714) has eigenvalues, $\lambda_{1}>0$ and $\lambda_{2}<0$, and the second x-braced lattice (*k* = 1.0834) produces eigenvalues, $\lambda_{1}\approx 0$ and $\lambda_{2}\approx 0$. We verify the polarization behavior of the second x-braced lattice, with repeating eigenvalues that are approximately zero, by applying an arbitrary Raleigh mode deformation in Equation (1) or Equations (12–13). So, instead of using the required polarization vector h, with b=0.7677 and c=0.6408, for constructing the solution to the zero eigenvalue, we apply a random polarization, with b=0.5139 and c=0.8579, to both lattices and calculate their nodal displacements. The deformed shape of the two x-braced lattices can be seen in Figure 12, showing that displacements propagate in the first lattice because one of its eigenvalues is not zero. However, in the second lattice, we see that although displacements are not blocked at n=0, they propagate one unit cell distance further and then get completely blocked at n=1. Therefore, the second x-braced lattice can polarize an arbitrary Raleigh wave into a polarized Raleigh wave. The polarization behavior can be checked by simply deriving the required polarization vector from the calculated displacements at n=1 and comparing it with the analytical solution by Equation (12) or (13).

**Figure 13:**
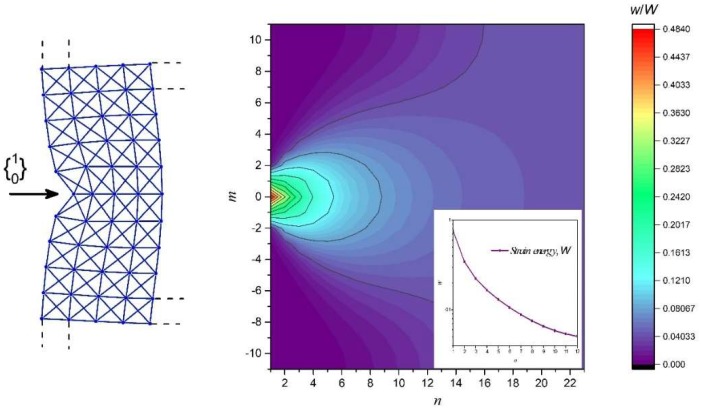
A contour map of normalized strain energy distribution of an x-braced lattice having k=0.93 with dimensions, m=22 and n=22, under a point load, $f_{0m}=\left\{\begin{array}{c} 1\\ 0 \end{array}\right\}\delta_{m0}$. The inset shows the total strain energy in a vertical layer of unit cell thickness vs. the lattice index, n.

Lastly, we consider a natural boundary condition, where $f_{0m}=\left\{\begin{array}{c} 1\\ 0 \end{array}\right\}\delta_{m0}$**,** which describes a point load or an indentation force acting at the mid-point of the x-braced lattice with a stiffness parameter, k=0.93, and a lattice vertical dimension, *M* = 22. The solution was constructed from Equations (14), (20), and (21), and in Figure 13, we show the lattice deformation configuration and the normalized strain energy distribution contour map. However, the x-braced lattice in this case experiences a very slow decay in deformation and strain energy compared to its boundary as deformation at a node would be composed of all possible static Raleigh modes. As we move along the lattice, *n,* it can be realized that the strain energy at the boundary is much more concentrated, and moving away from the boundary, it begins to take a Gaussian form till the energy distribution becomes rather uniform in the material interior.

The examples shown in this paper can be modelled using a commercial finite element analysis software package such as ANSYS, where the top and bottom edges of the lattice are constrained to have only horizontal displacements, rendering a seamless cyclic model [24]. Comparing deformations from the above examples to their model solutions from ANSYS, the difference was of the order, 10^−6^, which shows the high accuracy of our analytical results.

## 9. Conclusions

In this paper, we have discussed in detail the 2DoF general Raleigh wave mode solutions for analyzing essential boundary and natural boundary conditions. The concept of bandgap design for deformation blockage and achieving the RSV effect was also introduced. Such bandgap analysis has been shown to be readily applicable to any fundamental Raleigh wave solution due to the solution dependence on a single zero eigenvalue (λ=0) corresponding with the Fourier parameter, *q*. The bandgap relationships presented can serve as tools for programming the unit-cell aspect ratio and stiffness parameter of an x-braced lattice to block a specific static Raleigh mode or filter out irrelevant modes when an applied boundary condition is a combination of several modes. The case of repeated zero eigenvalues has also been shown to present a unique class of nonlocal lattice that can serve as polarizers to induce blockage at n=1 of an arbitrarily polarized Raleigh wave. Solutions to non-Raleigh mode boundary conditions were shown to depend on all possible Fourier modes with controllable decay parameters, providing an opportunity to program the overall strain energy distribution in the material sample. An equivalent continuum theory [36], analysis of strain energy spectral density, and information entropy of deformation [37] for nonlocal mechanical metamaterials can be an interesting separate study for the future.

An understanding of the methodologies presented in this study can be key in driving future research on RSV metamaterials, where uniqueness in deformation and strain energy distribution patterns are harnessed to design smart materials and structures with interesting functionalities and properties, such as load pattern recognition, high resilience, and stress alleviation.

## Appendix A

Finding eigenvectors of the transfer matrix, H(q), of 2DoF x-braced lattice:

(A1) $$H\left(q\right)=\left[\begin{array}{cccc} 0 & 0 & 1 & 0\\ 0 & 0 & 0 & 1\\ \beta_{1} & \beta_{3}i & \beta_{4} & \beta_{6}i\\ \beta_{2}i & \beta_{1} & \beta_{5}i & \beta_{7} \end{array}\right]$$

$$\beta_{1}=-\frac{\left(\sqrt{2\;}\cos q+k\cos2q\right)}{k+\sqrt{2}\cos q},\;\;\beta_{2}=\frac{2\left(\sqrt{2\;}+k\cos q\right)\sin q}{k+\sqrt{2}\cos q},\;\;\beta_{3}=\frac{k\;\sin q}{k+\sqrt{2}\cos q},\;\;\beta_{4}=\frac{2\left(\sqrt{2\;}+k\right)\cos q}{k+\sqrt{2}\cos q}$$

$$\beta_{5}=-\frac{2\left(\sqrt{2}+k\right)\sin q}{k+\sqrt{2}\cos q},\;\;\beta_{6}=-\frac{2\left(k-\sqrt{2\;}\left(k\cos q-1\right)\right)\sin q}{k+\sqrt{2}\cos q},\;\;\beta_{7}=\;\frac{\left(2+k\sqrt{2}-2\cos q\right)\left(2+\sqrt{2\;}k\cos q\right)}{k+\sqrt{2}\cos q}$$

To find eigenvectors, $\left\{\begin{array}{c} h\left(q\right)\\ \lambda h\left(q\right) \end{array}\right\}=\left\{\begin{array}{c} \begin{array}{c} x\\ y\\ w \end{array}\\ z \end{array}\right\}$, of **H** (*q*, we use the row reduction echelon method to solve (H(q)−Iλ)h(q)=0 and get the equation:

(A2) $$\left[\begin{array}{cccc} 1 & 0 & \frac{1}{-\lambda } & 0\\ 0 & 1 & 0 & \frac{1}{-\lambda }\\ 0 & 0 & 1 & \frac{\beta_{6}i+\beta_{3}i\frac{1}{\lambda }}{\left(\beta_{4}-\lambda \right)+\beta_{1}\frac{1}{\lambda }}\\ 0 & 0 & 0 & \left(\beta_{7}+\beta_{1}\frac{1}{\lambda }\right)-\left(\beta_{5}i+\beta_{2}i\frac{1}{\lambda }\right)\frac{\beta_{6}i+\beta_{3}i\frac{1}{\lambda }}{\left(\beta_{4}-\lambda \right)+\beta_{1}\frac{1}{\lambda }} \end{array}\right]\left\{\begin{array}{c} \begin{array}{c} x\\ y\\ w \end{array}\\ z \end{array}\right\}=\left\{\begin{array}{c} \begin{array}{c} 0\\ 0\\ 0 \end{array}\\ 0 \end{array}\right\}$$

Writing down equations, *z* is 0, which makes the eigenvector zero, but since an eigenvector cannot be zero, we take *z* as any real or complex number. However, taking *z* = 1 and solving for (*x*, *y*, *w*, *z*) we get:

(A3) $$\left\{\begin{array}{c} h\left(q\right)\\ \lambda \left(q\right)h\left(q\right) \end{array}\right\}=C\left\{\begin{array}{c} \begin{array}{c} \frac{i\left(\beta_{3}+\beta_{6}\lambda \right)}{\lambda \left(\lambda^{2}-\beta_{4}\lambda -\beta_{1}\right)}\\ \frac{1}{\lambda }\\ \frac{i\left(\beta_{3}+\beta_{6}\lambda \right)}{\lambda^{2}-\beta_{4}\lambda -\beta_{1}} \end{array}\\ 1 \end{array}\right\}$$

The above expression can be rewritten as:

(A4) $$\left\{\begin{array}{c} h\left(q\right)\\ \lambda \left(q\right)h\left(q\right) \end{array}\right\}=C\left\{\begin{array}{c} \begin{array}{c} i\left(\beta_{3}+\beta_{6}\lambda \right)\\ \lambda^{2}-\beta_{4}\lambda -\beta_{1}\\ i\;\lambda \left(\beta_{3}+\beta_{6}\lambda \right) \end{array}\\ \lambda \left(\lambda^{2}-\beta_{4}\lambda -\beta_{1}\right) \end{array}\right\}$$

Constructing real-valued cyclic Raleigh wave solutions:

*Complex Eigenvalues:*

$$h\left(q\right)=\left\{\begin{array}{c} a\pm ib\\ -c+id \end{array}\right\}:$$

(A5) $$d_{nm}^{\left(1\right)}=C_{1}\rho^{n}\left(q\right)\left\{\left\{\begin{array}{c} a\;\cos\;\left(\theta n+qm\right)-b\;\sin\;\left(\theta n+qm\right)\\ c\;\cos\left(\theta n+qm\right)-d\;\sin\;\left(\theta n+qm\right) \end{array}\right\}+i\left\{\begin{array}{c} a\;\sin\;\left(\theta n+qm\right)+b\;\cos\;\left(\theta n+qm\right)\\ c\;\sin\;\left(\theta n+qm\right)+d\;\cos\;\left(\theta n+qm\right) \end{array}\right\}\right\}$$

(A6) $$d_{nm}^{\left(2\right)}=C_{1}\rho^{n}\left(q\right)\left\{\left\{\begin{array}{c} a\;\cos\;\left(-\theta n+qm\right)+b\;\sin\;\left(-\theta n+qm\right)\\ -c\;\cos\;\left(-\theta n+qm\right)-d\;\sin\;\left(-\theta n+qm\right) \end{array}\right\}+i\left\{\begin{array}{c} a\;\sin\;\left(-\theta n+qm\right)-b\;\cos\;\left(-\theta n+qm\right)\\ -c\;\sin\;\left(-\theta n+qm\right)+d\;\cos\;\left(-\theta n+qm\right) \end{array}\right\}\right\}$$

Possible cyclic harmonic solutions are obtained by summing and subtracting the corresponding real and imaginary parts of the above equations as shown below:

(A7) dnm=Re dnm(1)+Re dnm(2)=Cρn(q){a cos qm−d sin qm}

(A8) dnm=Im dnm(1)−Im dnm(2)=Cρn(q){b cos qmc sin qm}

*Real Eigenvalues:*

Case 1:

$$h\left(q\right)=\left\{\begin{array}{c} ib\\ c \end{array}\right\}$$

(A9) $$d_{nm}=C_{2}\lambda^{n}\left(q\right)\left\{\left\{\begin{array}{c} -b\;\sin\;qm\\ c\;\cos\;qm \end{array}\right\}+i\left\{\begin{array}{c} b\;\cos\;qm\\ c\;\sin\;qm \end{array}\right\}\right\}$$

The real-cyclic solution is the imaginary part of the solution above:

(A10) $$d_{nm}=C_{2}\lambda^{n}\left(q\right)\left\{\begin{array}{c} b\;\cos\;qm\\ c\;\sin\;qm \end{array}\right\}$$

Case 2:

$$h\left(q\right)=\left\{\begin{array}{c} b\\ ic \end{array}\right\}$$

(A11) $$d_{nm}=C_{2}\lambda^{n}\left\{\left\{\begin{array}{c} b\;\cos\;qm\\ -c\;\sin\;qm \end{array}\right\}+i\left\{\begin{array}{c} b\;\sin\;qm\\ c\;\cos\;qm \end{array}\right\}\right\}$$

The real-cyclic solution is the real part of the solution above:

(A12) $$d_{nm}=C_{2}\lambda^{n}\left(q\right)\left\{\begin{array}{c} b\;\cos\;qm\\ -c\;\sin\;qm \end{array}\right\}$$

Finding the zero-eigenvalue relationship for a 2DoF x-braced lattice:

(A13) $$\det\;H\left(q\right)=\prod_{i=1}^{n}\lambda_{i}\left(q\right)=\frac{k{\left(k+\sqrt{2}\cos q\right)}^{2}}{k{\left(k+\sqrt{2}\cos q\right)}^{2}}$$

Since λ(q)=0 when det H(q)=0, we write the zero-eigenvalue relationship as:

(A14) $$k+\sqrt{2}\cos q=0$$

The transfer matrix, H(q), when we introduce aspect ratio, α:

(A15) $$H\left(q\right)=\left[\begin{array}{cccc} 0 & 0 & 1 & 0\\ 0 & 0 & 0 & 1\\ \gamma_{1} & \gamma_{3}i & \gamma_{4} & \gamma_{6}i\\ \gamma_{2}i & \gamma_{1} & \gamma_{5}i & \gamma_{7} \end{array}\right]$$

$$\gamma_{1}=-\frac{{\left(1+\alpha^{2}\right)}^{2}\cos q+2k\alpha^{3}\sqrt{1+\alpha^{2}}\cos2q}{2k\alpha^{3}\sqrt{1+\alpha^{2}}+{\left(1+\alpha^{2}\right)}^{2}\cos q},\;\;\gamma_{2}=\frac{2\alpha \left({\left(1+\alpha^{2}\right)}^{2}+2k\alpha^{3}\sqrt{1+\alpha^{2}}\cos q\right)\sin q}{2k\alpha^{3}\sqrt{1+\alpha^{2}}+{\left(1+\alpha^{2}\right)}^{2}\cos q}$$

$$\gamma_{3}=\frac{4k\alpha^{2}\sqrt{1+\alpha^{2}}\cos q\;\sin q}{2k\alpha^{3}\sqrt{1+\alpha^{2}}+{\left(1+\alpha^{2}\right)}^{2}\cos q},\;\;\gamma_{4}=\frac{2\left(1+\alpha^{2}\left(2+\alpha^{2}+2k\alpha \sqrt{1+\alpha^{2}}\right)\right)\cos q}{2k\alpha^{3}\sqrt{1+\alpha^{2}}+{\left(1+\alpha^{2}\right)}^{2}\cos q}$$

$$\gamma_{5}=-\frac{2\alpha (1+\alpha^{2}(2+\alpha^{2}+2k\alpha \sqrt{1+\alpha^{2}}))\sin q}{2k\alpha^{3}\sqrt{1+\alpha^{2}}+{\left(1+\alpha^{2}\right)}^{2}\cos q},\;\;\gamma_{6}=\frac{2\alpha \left(-2k\sqrt{1+\alpha^{2}}-{\left(1+\alpha^{2}\right)}^{2}+{\left(1+\alpha^{2}\right)}^{2}\cos q\right)\sin q}{2k\alpha^{3}\sqrt{1+\alpha^{2}}+{\left(1+\alpha^{2}\right)}^{2}\cos q}$$

$$\gamma_{7}=-\frac{\sqrt{1+\alpha^{2}}\left({\left(1+\alpha^{2}\right)}^{3/2}+2k\alpha^{3}\cos q\right)\left(-2k-{\left(1+\alpha^{2}\right)}^{3/2}+{\left(1+\alpha^{2}\right)}^{3/2}\cos q\right)}{k\left(2k\alpha^{3}\sqrt{1+\alpha^{2}}+{\left(1+\alpha^{2}\right)}^{2}\cos q\right)}$$

Finding the total strain energy along the index, *n*:

(A16) $$w=\frac{1}{2}\sum_{n^{\prime }m^{\prime }}d_{n^{\prime }m^{\prime }}^{*}k_{n-n^{\prime }m-m^{\prime }}d_{n^{\prime }m^{\prime }}$$

(A17) $$W=\frac{1}{2}\sum_{M}\sum_{n^{\prime }m^{\prime }}d_{n^{\prime }m^{\prime }}^{*}k_{n-n^{\prime }m-m^{\prime }}d_{n^{\prime }m^{\prime }}$$
